# Birth by Caesarean section and otitis media in childhood: a retrospective cohort study

**DOI:** 10.1038/s41598-020-62229-y

**Published:** 2020-03-23

**Authors:** Maria Hartley, Christy G. Woolcott, Joanne M. Langley, Mary M. Brown, Jillian Ashley-Martin, Stefan Kuhle

**Affiliations:** 1 Perinatal Epidemiology Research Unit, Depts of Obstetrics & Gynaecology and Pediatrics, Dalhousie University, Halifax, NS Canada; 2 Department of Pediatrics, Dalhousie University, Halifax, Nova Scotia Canada

**Keywords:** Epidemiology, Paediatric research

## Abstract

The objective of the present study was to examine the association between birth by Caesarean section (CS) and otitis media (OM) in childhood. We assembled a retrospective cohort of children born between 2003 and 2007 in Nova Scotia and followed them through to 2014. The cohort was derived through a linkage of the Nova Scotia Atlee Perinatal Database with provincial administrative health data. Cox proportional hazards, negative binomial regression and logistic regression were used to examine the association between CS and OM. Among the 36,318 children, 27% were born by CS, and 78% had at least one OM episode (median 2 episodes). Children born by CS were at a slightly higher risk of OM (hazard ratio 1.06, 95% confidence interval (CI) 1.03, 1.09), had more OM episodes in the first 7 years of life (incidence rate ratio 1.04, 95% CI 1.01, 1.07), and were more likely to be above the 95th percentile for OM episodes than children born vaginally (odds ratio 1.10, 95% CI 0.99, 1.23). Our study shows that birth by CS is weakly associated with OM in childhood, but the clinical and public health impact of these findings is small.

## Introduction

Birth by Caesarean section (CS) is associated with an increased risk of childhood conditions such as allergic disorders, diabetes, and obesity^1,2^. These associations are hypothesized to arise, in part, because birth by CS may promote adverse changes in the gut microbiome relative to vaginal birth. These changes may impair the normal functioning of the immune system and thereby contribute to the development of immune-related disorders^3,4^. Otitis media (OM) is a common infection of the middle ear affecting more than 80% of children^5^. It is the second most common reason for primary care visits and the leading cause of childhood antibiotic use and temporary hearing loss^5–8^. Considering that CS and OM are associated with many conditions in common, such as obesity and allergic disorders^6,9,10^, an association between CS and OM is biologically plausible.

Of the three that studies have examined the association between CS and OM so far, two reported a positive association^11,12^, while the other did not find an association^13^. Two of the studies were limited by small, selected samples and were published in the 1980s and 1990s, respectively^11,13^. We therefore sought to examine the association between birth by CS and health care use for OM in childhood in a large population-based sample in the Canadian province of Nova Scotia.

## Methods

We used data from a retrospective cohort of children born between 2003 and 2007 to mothers residing in the Canadian province of Nova Scotia at the time of birth and followed through to 2014; multiples and children with major congenital anomalies were excluded. The cohort was derived through a linkage of the Nova Scotia Atlee Perinatal Database with provincial administrative health data. Children were included in the present analysis if they were born at 37 weeks’ gestation or later and had at least two months of follow-up in the administrative health databases. The study was approved by the IWK Health Centre Research Ethics Board (File #1015756), the Health Data Nova Scotia Data Access Committee, and the Reproductive Care Program Joint Data Access Committee. The need for informed consent for this database-based study was waived by the IWK Ethics Board as per the Nova Scotia Personal Health Information Act. All procedures performed were in accordance with the ethical standards of the institutional research committee and with the *Tri-Council Policy Statement: Ethical Conduct for Research Involving Humans, December 2014*.

The Nova Scotia Atlee Perinatal Database collects data from all pregnancies resulting in births to infants >500 g or >20 weeks’ gestation to residents of Nova Scotia since 1988; it includes information regarding demographics, procedures, interventions, diagnoses, morbidity, and mortality of the mother and infant. The physician billing database contains all services rendered by a physician and insured by the provincial medical insurance plan (Medical Services Insurance). The Canadian Institute for Health Information Discharge Abstract Database contains demographic and clinical information for hospital discharges. The Insured Patient Registry tracks data on all beneficiaries of the provincial medical insurance plan and was used to determine if and when individuals within the cohort left the province or died. The perinatal database and the administrative databases were linked via the health card number, a unique identifier that is assigned at birth to each beneficiary of the provincial medical insurance plan.

### Outcome

The primary outcome was time to the first physician visit or hospital stay with an International Classification of Diseases (ICD) code for non-suppurative or suppurative OM (ICD-9: 381–382; ICD-10-CA: H65–66) after two months of age. Secondary outcomes were the number of OM episodes (an episode was defined as a visit with a code for OM and any subsequent visits within a 30-day period) and being above the 95th percentile of the number of OM episodes (high utilizer) until the age of 7 years.

### Exposure

The main exposure of interest was mode of delivery, categorized as vaginal delivery or CS. For a secondary analysis, CS was further broken down into CS before the second stage of labor, which includes both planned CS done before the initiation of labor and CS done in the first stage of labor, and CS during the second stage of labor to capture the likelihood of the fetus’ exposure to the vaginal flora prior to delivery by CS. Vaginal delivery included both unassisted and assisted vaginal deliveries.

### Confounding variables

**Figure 1:**
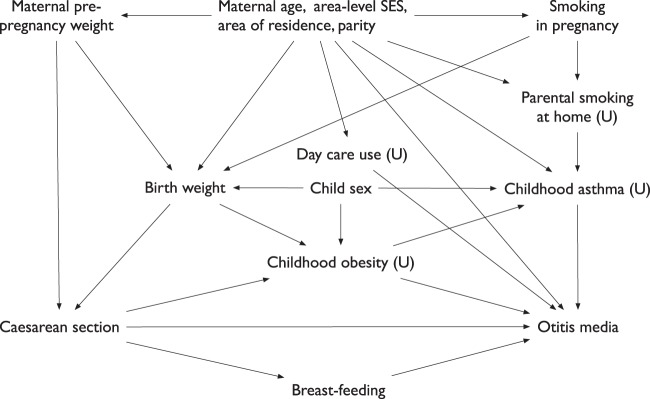
Directed acyclic graph with the hypothesized relationships between birth by Caesarean section and otitis media. Abbreviations: *SES* socioeconomic status, *U* unmeasured.

We identified confounding variables a priori using a directed acyclic graph (Fig. 1). These included maternal pre-pregnancy weight (not overweight or obese, overweight, and obese), birth weight for gestational age (small for gestational age [<10th percentile], appropriate for gestational age [10th to 90th percentile], or large for gestational age [>90th percentile] as per the Canadian growth ref. ^14^), maternal smoking during the pregnancy (any smoking at the first prenatal visit or on admission to the labor ward was considered smoking during pregnancy), area-level income quintile, area of residence (rural vs. urban, based on Canadian postal codes), sex, parity (categorized as 0, 1, 2, and ≥3 excluding the current pregnancy), and maternal age at delivery.

### Statistical analysis

Descriptive statistics of the cohort were summarized by mode of delivery. Cox proportional hazards regression was used to examine the association between CS and time to the first OM episode until the end of their medical insurance coverage or the end of the study period (Dec 31, 2014), whichever came first. The Efron approximation was used for ties. The proportionality assumption was assessed using Schoenfeld residuals. The association between CS and the number of OM episodes until the age of 7 years (i.e. the latest follow-up timepoint that is available for all children in the cohort) was examined by negative binomial regression. Logistic regression was used to model the association between CS and being above the 95th percentile of number of OM episodes. Only children with complete follow-up for the full study period (n = 32,489) were included in the latter two analyses. Models were adjusted for the confounding variables identified in the directed acyclic graph. Multiple imputation with chained equations (10 iterations, 20 imputed datasets) was used to impute missing values of the model covariates. All statistical analyses were performed using R version 3.5^15^ and RStudio^16^.

## Results

Of the 42,050 singleton infants born to Nova Scotian mothers between January 1, 2003 to December 31, 2007, 39,369 could be linked with administrative health data and had non-missing and plausible birth weight and gestational age information. After exclusion of children who were born preterm (n = 2960), had missing data on the exposure (n = 32), had less than two months of follow-up due to death or migration out of the province (n = 59), the analysis sample consisted of 36,318 children.

Twenty-seven percent of children in the sample were born by CS (21% before and 6% at the second stage of labor). The sociodemographic and clinical characteristics of the children by mode of delivery are shown in Table 1. Compared to children delivered vaginally, children delivered by CS had mothers who, on average, were older, had higher pre-pregnancy weight, were less likely to smoke, and were more likely to be married or in a common-law partnership, and have gestational diabetes; children delivered by CS were also more likely to be large for gestational age.

**Table 1:** Sociodemographic and clinical characteristics of Nova Scotia women with a singleton birth between 2003 and 2007, stratified by mode of delivery (N = 36,318).

	Vaginal delivery	Caesarean section
	n = 26,589 (73.2%)	n = 9729 (26.8%)
Maternal age, years (mean, SD)	28 (5.6)	30 (5.4)
Area-level income quintile (%)		
Q1 (lowest)	20	19
Q2	21	21
Q3	22	21
Q4	21	22
Q5 (highest)	16	16
Missing	0.4	0.3
Rural residence (%)	30	28
Married or common-law (%)	66	73
Parity (%)		
0	44	48
1	36	37
2	14	11
≥ 3	6.4	3.5
Maternal pre-pregnancy weight status (%)		
Not overweight, not obese	47	36
Overweight	19	20
Obese	17	26
Missing	18	18
Gestational diabetes mellitus (%)	2.7	5.3
Smoking during pregnancy (%)		
Yes	29	25
Missing	0.8	0.7
Male offspring sex (%)	50	53
Birth weight for gestational age (%)		
Appropriate (10th–90th percentile)	79	72
Small (<10th percentile)	7.1	6.9
Large (>90th percentile)	14	21

Abbreviations: Q quintile, SD standard deviation.

Seventy-eight percent of children used health services for OM. Among these, the median time until first health service contact for OM was 491 days (interquartile range [IQR] 310–966), and the median number of OM episodes was 2 (IQR 1–6). The 95th percentile of OM episodes was 17 visits.

Table 2 shows the results from the regression analysis for the main exposure. In the adjusted models, children born by CS were more likely to have had used health services for OM (hazard ratio 1.06, 95% confidence interval (CI) 1.03, 1.09), had more OM episodes (incidence rate ratio 1.04, 95% CI 1.01, 1.07), and were more likely to be above the 95th percentile for OM episodes than children born vaginally (odds ratio 1.10, 95% CI 0.99, 1.23).

**Table 2:** Association between mode of delivery and otitis media in the offspring.

	Vaginal delivery	Caesarean section
**Risk of otitis media**		
Time to first episode, median (IQR) [days]	495 (309–972)	486 (312–951)
Unadjusted HR (95% CI)	Ref.	1.09 (1.06, 1.12)
Adjusted HR (95% CI)	Ref.	1.06 (1.03, 1.09)
**Number of otitis media episodes**		
Median (IQR)	2 (1–5)	2 (1–6)
Unadjusted IRR (95% CI)	Ref.	1.11 (1.07, 1.14)
Adjusted IRR (95% CI)	Ref.	1.04 (1.01, 1.07)
High utilizer* for otitis media episodes		
%	5.0%	6.2%
Unadjusted OR (95% CI)	Ref.	1.25 (1.12, 1.39)
Adjusted OR (95% CI)	Ref.	1.10 (0.99, 1.23)

An episode of otitis media was defined as a physician visit or hospital stay with an ICD code for non-suppurative or suppurative otitis media and any subsequent visits within a 30-day period.

*Number of episodes >95th percentile.

Abbreviations: *CI* confidence interval, *HR* hazard ratio, *IQR* interquartile range, *IRR* incidence rate ratio, *OR* odds ratio.

The unadjusted and adjusted mean difference in the number of OM episodes between the CS and vaginal birth groups was 0.47 (95% CI 0.32, 0.62) and 0.11 (95% CI 0.02, 0.20), respectively.

Table 3 shows the results from the regression analysis when CS was further split by stage of labor. The magnitude of the associations was similar between children born by CS before or during the second stage of labor.

**Table 3:** Association between timing of Caesarean section and otitis media in the offspring.

	Vaginal delivery	Caesarean section before second stage of labor	Caesarean section during second stage of labor
**Risk of otitis media**			
Time to first episode, median (IQR) [days]	495 (309–972)	481 (308–937)	506 (327–983)
Unadjusted HR (95% CI)	Ref.	1.09 (1.06, 1.13)	1.09 (1.03, 1.14)
Adjusted HR (95% CI)	Ref.	1.06 (1.03, 1.09)	1.06 (1.01, 1.12)
**Number of otitis media episodes**			
Median (IQR)	2 (1–5)	3 (1–6)	3 (1–6)
Unadjusted IRR (95% CI)	Ref.	1.10 (1.07, 1.14)	1.12 (1.06, 1.19)
Adjusted IRR (95% CI)	Ref.	1.04 (1.01, 1.08)	1.02 (0.96, 1.08)
High utilizer* for otitis media episodes			
%	5.0%	6.1%	6.3%
Unadjusted OR (95% CI)	Ref.	1.24 (1.11, 1.39)	1.27 (1.04, 1.54)
Adjusted OR (95% CI)	Ref.	1.10 (0.98, 1.24)	1.09 (0.89, 1.34)

An episode of otitis media was defined as a physician visit or hospital stay with an ICD code for non-suppurative or suppurative otitis media and any subsequent visits within a 30-day period.

*Number of episodes> 95th percentile

Abbreviations: *CI* confidence interval, *HR* hazard ratio, *IQR* interquartile range, *IRR* incidence rate ratio, *OR* odds ratio.

## Discussion

Results from this population-based retrospective cohort showed that compared to vaginal birth, CS delivery is associated with a slightly higher risk of OM and a higher number of OM episodes in the offspring in the first seven years of life compared to vaginal delivery, but the effect is small. The observed association did not differ between children born by CS before or during the second stage of labor.

Birth by CS may be associated with the development of OM in childhood through two pathways. Firstly, there is evidence from laboratory, clinical, and epidemiological studies that CS is associated with changes in the gut microbiome compared to vaginal delivery^17^. These changes adversely affect the development of the immune system and increase the risk of immune-related conditions such as allergic disorders, asthma, diabetes, and celiac disease in the offspring^2–4^. Allergic disorders and asthma in turn are known risk factors for OM^6,10^. A second potential pathway involves the association between CS and childhood obesity^1^ which is hypothesized to be due to alterations in the gut microbiota following CS that lead to increased energy harvesting from the gut. We and others have previously described an association between childhood obesity and OM^9,18,19^. Increased levels of inflammatory markers, dysfunction of the Eustachian tube due to peritubal fat accumulation, gastroesophageal reflux, and a dysfunction of the taste nerve have been proposed as underlying causes for this association^20^.

Three previous studies have examined the association between mode of delivery and the risk of having OM. In 1986, Mansueti *et al*. compared perinatal and neonatal characteristics of 141 children with tympanostomy tubes born at a French hospital to those of children without tympanostomy tubes^11^. The odds ratio for having tympanostomy tubes as a treatment for at least one previous OM episode among children born by CS compared to those born vaginally was 2.23 (95% CI 1.49, 3.34); an adjusted analysis was not performed. Mansfield *et al*. used hospital records of 284 three-year-old children born in two hospitals in North Carolina in 1987 to examine if CS was a risk factor for the occurrence of OM^13^. They found no evidence for an association between CS and OM in the offspring (unadjusted relative risk 1.12, 95% CI 0.88, 1.44).

The most recent study, published in 2018 by Kørvel-Hanquist *et al*.^12^, assessed risk factors for childhood OM in a cohort of more than 50,000 mother-child pairs^21^. The authors reported adjusted odds ratios of 1.20 (95% CI 1.07, 1.35) and 1.23 (95% CI 1.13, 1.33) for having > 3 episodes of OM at ages 18 months and 7 years, respectively, among children born by CS compared to children born vaginally. However, the estimates from this study may be biased as the multivariable regression models were solely built based on statistical significance and did not consider causal relationships between variables^22^. The study was further limited by the reliance on the mother’s recall of OM episodes and a loss to follow-up of 45% at age 18 months.

The magnitude of the association in the present study was similar to that reported by Mansfield^13^. A 6% higher hazard of OM and 4% higher number of OM episodes (corresponding to on average 0.11 more OM episodes in a child born by CS) at age 7 years in children born by CS compared to children born vaginally is clinically not meaningful as it would not influence the decision to perform a CS. From a public health perspective, the potential for reducing the economic burden of OM is likewise negligible since, despite overuse of the procedure, a fair proportion of CS in Western countries are medically indicated and cannot be avoided^23^.

The strengths of the current study are the use of a large, population-based sample with a long follow-up, the ability to control for a broad range of confounding variables, and the use of physician-based diagnoses of OM as opposed to parent-report. However, diagnostic codes in administrative health data may rather reflect billing practices than medical conditions and symptomology. The incidence of OM in our study (533 per 1000 person-years) is difficult to conclusively compare to the incidence reported in other studies, as estimates vary considerably depending on the methodology, setting, and follow-up period from 121 per 1000 person-years before age 5 years to 1.8 visits per year in the first 24 months of life^24–27^.

## Conclusions

We have shown in a large population-based sample from the Canadian province of Nova Scotia that children born by CS have a slightly higher risk of OM and a higher number of OM episodes in the first 7 years of life compared to those born vaginally. However, the associations were weak and only of limited clinical and public health significance.

## Data Availability

The data that support the findings of this study are available from the Reproductive Care Program of Nova Scotia and Health Data Nova Scotia, Canada. As these data constitute personal health information, they can only be accessed from within Nova Scotia as per provincial privacy laws. Researchers must submit a data access application to the database custodians and upon approval sign a data sharing agreement (http://rcp.nshealth.ca/atlee-database/data-access and https://medicine.dal.ca/departments/department-sites/community-health/research/hdns/data-access-guidelines.html, respectively).
